# A phased antenna array for surface plasmons

**DOI:** 10.1038/srep25037

**Published:** 2016-04-28

**Authors:** Dirk Jan W. Dikken, Jeroen P. Korterik, Frans B. Segerink, Jennifer L. Herek, Jord C. Prangsma

**Affiliations:** 1 Optical Sciences, MESA^+^ Institute for Nanotechnology, University of Twente, P.O. Box 217, 7500AE Enschede, The Netherlands

## Abstract

Surface plasmon polaritons are electromagnetic waves that propagate tightly bound to metal surfaces. The concentration of the electromagnetic field at the surface as well as the short wavelength of surface plasmons enable sensitive detection methods and miniaturization of optics. We present an optical frequency plasmonic analog to the phased antenna array as it is well known in radar technology and radio astronomy. Individual holes in a thick gold film act as dipolar emitters of surface plasmon polaritons whose phase is controlled individually using a digital spatial light modulator. We show experimentally, using a phase sensitive near-field microscope, that this optical system allows accurate directional emission of surface waves. This compact and flexible method allows for dynamically shaping the propagation of plasmons and holds promise for nanophotonic applications employing propagating surface plasmons.

If one sets out to control the flow of light on the smallest possible scale, surface plasmon polaritons (SPPs) are a natural choice since their lateral confinement to the surface by far exceeds the confinement possible with dielectric structures^1^. To control the flow of surface plasmons, many advances have been made over the years by structuring the metal surface to create e.g. waveguides, prisms, lenses and Bragg mirrors, for SPP’s^2^,^3^,^4^,^5^. It all starts, however, with launching SPPs on the metal surface. To achieve this with a small footprint and with control over directionality, gratings^6^,^7^,^8^,^9^,^10^ and two dimensional hole arrays^11^,^12^ are used. It has been shown that a limited number of well-positioned holes can give directional SPP emission^13^,^14^,^15^,^16^ and even one hole^17^ already gives a dipolar SPP radiation pattern. Strategies based on patterns of holes in metal films are promising, however they are static in design, resulting in a fixed radiation pattern. Some control can be obtained by using the amplitude and phase distribution of a well-positioned focused beam on a hole array^18^; for further flexibility Gjonaj *et al.* explored the possibilities of spatial phase modulation for controlled launching of surface plasmons^19^,^20^,^21^,^22^. They used a large number of holes patterned in two dimensional arrays to generate SPPs whose phase was controlled using a spatial light modulator to enable, for instance, a miniaturized surface plasmon microscope.

Here we present a minimalistic approach in which a small number of holes are addressed individually and controlled in phase, allowing tunable directional emission of SPPs in a surface plasmon analogue of a phased antenna array^23^,^24^,^25^. The use of a phase sensitive near-field microscope enables us to make accurate measurements of the SPP interference, which we study in real and reciprocal space^26^. With our approach we show a clear advance for active control of the directional launching of surface plasmons and demonstrate that already a minimal number of SPP-sources are needed to obtain dynamic directional control. To illustrate the level of control obtained, we compare strategies using rationally chosen illumination phase patterns as well as a feedback controlled optimization of the signal detected by the near-field tip. In contrast to previous work on large hole arrays, our device is extremely small in footprint and has a low number of independent channels available for control, yet enables beams of SPPs that can be optimized to propagate in any arbitrary direction.

## Results and Discussion

### SPP distribution of a single hole-antenna

**Figure 1:**
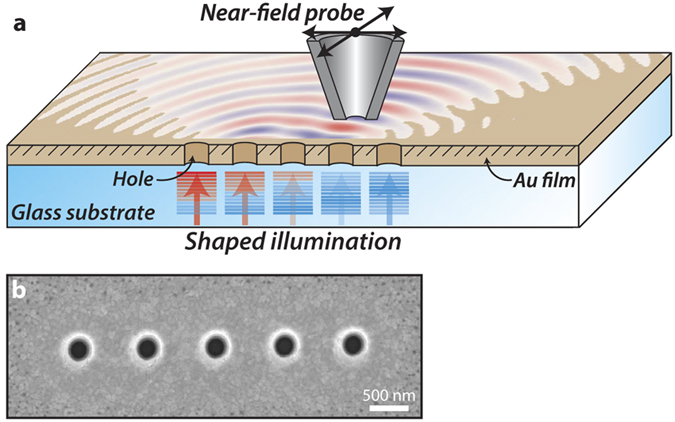
Hole-antenna system which has been made in a gold (Au) film. (**a**) schematic representation of the studied system containing a gold film with 5 holes which are illuminated from the bottom. The amplitude, phase and polarization state of each hole is controlled independently. The optical signal on the top surface is measured using a Near-field Scanning Optical Microscope. (**b**) shows a SEM micrograph of 5 holes in a 250 nm thick Au-film.

Figure 1 shows the schematic structure of the hole-antenna array and the method of illumination and detection. The structures are fabricated on a glass coverslip coated with a 250 nm thick film of gold. The individual hole-antennas have a diameter of 300 nm and have been made in arrays of 1, 2 and 5, which are illuminated from below with light from a continuous wave diode laser operating at *λ*_0_ = 950 nm, as illustrated schematically in Fig. 1. The hole size and film thickness are chosen such that no residual light from transmission through the film is present and that transmission of the holes is near cut-off of the lowest order mode. Hence, transmission through the film occurs via the fundamental mode of the hole; the corresponding near-field distribution of the fundamental mode is shown in Fig. S2 in the supplementary information. To visualize the propagating surface plasmons, a phase and polarization sensitive aperture type near-field scanning optical microscope (NSOM) is used, with which it is possible to extract amplitude, phase and polarization information of local fields near the metal-air interface^27^,^28^. More details on sample and setup can be found in the supporting information.

**Figure 2:**
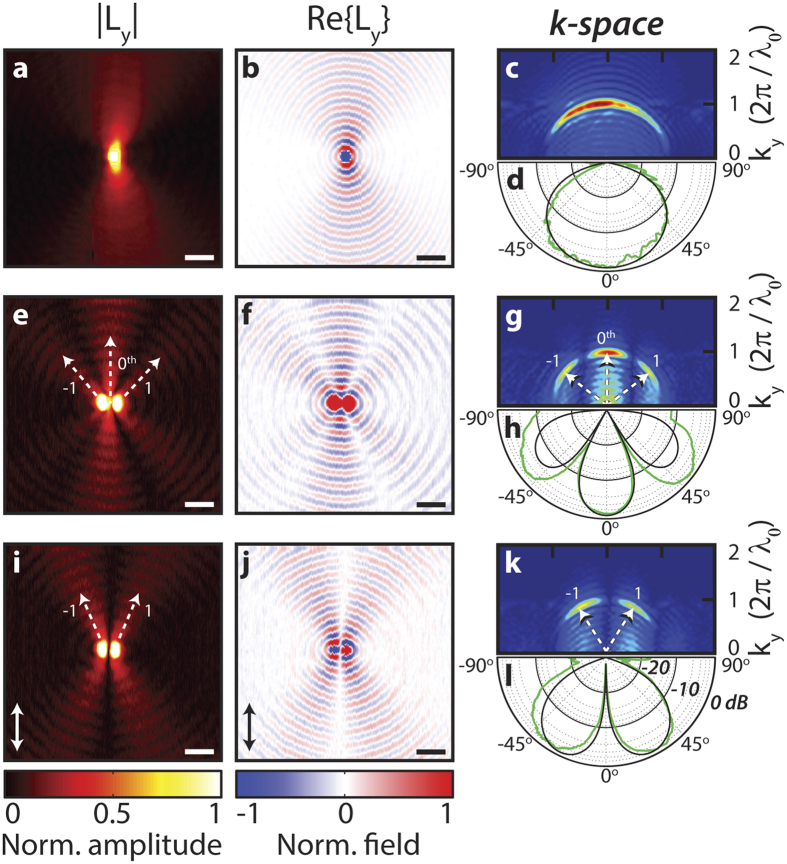
Near-field measurements on a single hole and two holes. (**a**,**e**,**i**) The absolute value of measured complex NSOM signal *L*_*y*_. (**b**,**f**,**j**) The real part of *L*_*y*_. (**c**,**g**,**k**) The absolute value of the 2D Fourier transform of *L*_*y*_, the k-vector distributions obtained. (**d**,**h**,**l**) Are polar plots of the SPP intensity on a logarithmic scale. These are obtained from the k-space distribution of both *L*_*x*_ and *L*_*y*_. The field distribution of a single hole is on the top row. For the two hole system the phase of the holes is in-phase (**e**–**h**) or half a cycle out-of-phase (**i**–**l**). Because our system is symmetric, the k-space image is only shown for positive values of *k*_*y*_. The scale bars represent a length of 2 *μ*m and the incident polarization was along the y-direction (indicated with the arrows in (**i**,**j**). In the polar plots the experimental data is plotted in green while the black lines show the modeled radiation patterns of the SPPs according to Equation 1. The hole spacing is 950 nm.

The absolute and real parts of the complex NSOM signal *L*_*y*_ of a single hole illuminated with linear polarization along the y-direction are shown in Fig. 2a,b. The amplitude is proportional to the square root of the SPP intensity and shows preferential emission in the y-direction, the real part also shows the phase of the propagating SPPs. By calculating the Fourier transform of the measured complex signal we obtain a two dimensional distribution of the k-vector of the electromagnetic field emerging from the single hole, see Fig. 2c. The high intensity at 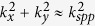 corresponds to the field components of propagating surface plasmon polaritons (SPPs). This reciprocal space image thus visualizes the SPP radiation pattern of a single hole. To make this clearer, in Fig. 2d we show the intensity of the detected signal at *k*_*spp*_ as a function of the azimuthal angle *θ* on a logarithmic scale. As expected^29^,^30^ a single hole illuminated with linear polarization along the y-direction shows great similarity to the radiation pattern of a magnetic dipole oriented along the x-direction indicated with the cos^2^(*θ*) curve in Fig. 2d, indicated in green.

Given that a single hole acts as dipolar source, we propose that a linear array of holes with controlled phase can act as a surface plasmon analogue of a phased antenna array. For such an array the radiation pattern as a function of the azimuthal angle *θ* can be written as:


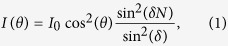


where 
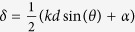
, *N* is the number of holes *I*_0_ is the intensity per hole, *d* is the hole spacing, *k* is the propagation constant (which in our case is equal to *k*_*spp*_) and *α* is the hole-to-hole phase difference. The cos^2^(*θ*) term in this equation originates from the dipolar nature of the SPP emission, the other term in the equation is simply the standard equation for a phased antenna array as can be found in textbooks^31^. For more details on the derivation of equation 1, we refer to the supplementary information.

### Directional control using a 2-element phased array

We first study the directional control obtained using an antenna array consisting of only 2 holes spaced by *λ*. This spacing is near the ultimate limit for which we can address phase and amplitude of individual holes due to the diffraction limit. We present here two extreme cases in which the phase of the holes is in-phase (Fig. 2e–h, *α* = 0) or half a cycle out-of-phase (Fig. 2i–l, *α* = *π*), achieved by addressing the illumination conditions of each individual hole using a spatial light modulator (SLM, see supporting information). The results in intensity (Fig. 2e,i) clearly show the ability to control the direction of SPP emission. In the direction perpendicular to the axis through both holes (*θ* = 0) for instance, the in-phase driving of the holes leads to a constructive interference, whereas the out-of-phase driving of the holes leads to destructive interference of SPPs and thus a minimum in intensity. The information contained in the phase sensitive signal Re(*L*_*y*_) in Fig. 2f,j shows the in- and out-of-phase behavior of the holes as well as the out of phase behavior of the neighboring diffraction orders. The reciprocal space images in Fig. 2g,k show the directional emission of the two holes. This is not only visible at *k*_*spp*_ for the surface plasmons but also within the light-line for the free space light as would be observed in a classical double slit experiment. Note that the relative contribution of SPPs and free space light in the Fourier space images depends on the scan area size used for the Fourier transformation, in this paper all Fourier space results are based on 15 × 15 *μ*m areas. The directional emission of the holes can be most clearly seen in the polar plots displayed in Fig. 2h,l. Here, the radiation pattern according to Equation 1 is superimposed on the experimental data, showing good qualitative agreement. Note that the model has no free parameters we can tune. Deviations are observed near the azimuthal angles of 90 degrees which we attribute to the measurement of quasi-cylindrical waves^32^ near the surface of the gold. The contribution of these non-surface-bound waves is most dominant under an angle *θ* of 90 degrees^33^. As they decay more rapidly than SPPs, the relative contribution in the k-space depends on the size of the scanned area in the experiment. Since our radiation patterns are based on this, measurements performed over smaller surfaces have a larger contribution of quasi-cylindrical waves whose effect is not included in the simple dipole model.

### Directional control with 5 holes

**Figure 3:**
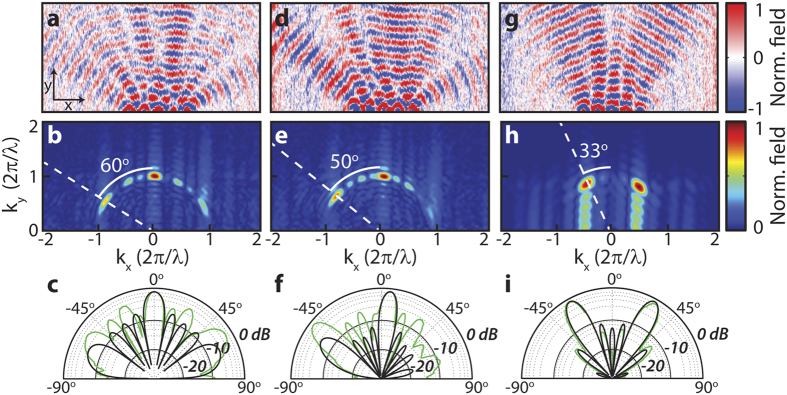
SPP radiation patterns of a hole-antenna system containing 5 holes, having a linear phase slope. (**a**–**c**) Zero phase slope, (**d**–**f**) consecutive holes have a phase difference of 0.22*π* and (**g**–**i**) consecutive holes have a phase difference of *π*. (**a**,**d**,**g**) Show the real part of *L*_*y*_. (**b**,**e**,**h**) Show the absolute value of the 2D Fourier transform of *L*_*y*_. (**c**,**f**,**i**) Are polar plots of the SPP intensity on a logarithmic scale. The experimental data is plotted in green while the black lines represent the modeled radiation patterns of the SPPs. The hole spacing is 950 nm as can be seen in the SEM micrograph in Fig. 1.

Control over directionality is further developed using a linear array of 5 holes, for which various phase slopes are programmed, this is very similar to a grating which is illuminated under different angles. The advantage of a larger array is simply that it allows for more angular resolution in the radiation pattern because it enables a higher resolution in k-space. Using the signal picked up by the near-field probe, the amplitude and phase conditions of each hole-antenna element is set manually. Figure 3 shows the SPP radiation patterns of a phased antenna array of 5 holes with three different phase relations. In panels a–c, all holes have equal phase. In d–f, consecutive holes have an arbitrarily chosen phase difference of 0.22*π*. In g–i, consecutive holes have a phase difference of *π*. From top to bottom, the three rows in Fig. 3 illustrate the real part of the complex signal *L*_*y*_, k-vector distribution and polar plots of the angular intensity of the SPPs. These radiation patterns illustrate that by applying a linear phase slope, the direction of the central peak (*θ* = 0°) can be shifted, in this case by 6° and 33°. For the largest phase slope this leads to the transition from a single peak dominated radiation pattern (a–c) to a two peak dominated radiation pattern (g–i). These two peaks in k-space are clearly visible in 3G as two SPP beams which symmetrically propagate outwards at *θ* = ±33°. The results of the model are shown in black. Especially Fig. 3i shows excellent agreement with the dipole model. Figure 3a,b show that in terms of the number of lobes and their direction, good agreement exists between experiment and the model, however the amplitudes show deviations. We attribute these differences to the experimental difficulty to address holes that are spaced close to the diffraction limit of our illumination optics. Indeed for holes spaced 3*λ* we found it much easier to achieve good agreement (See supporting information).

### Directional SPP emission using feedback controlled optimization

Finally we introduce an iterative optimization scheme, where the signal measured by the near-field tip is optimized. We show directional control of SPPs which can be programmed to propagate at any given angle. Using this approach it is no longer needed to manually set the correct illumination conditions of each hole in order to obtain control on directionality, as was done in Fig. 3. For this approach we use the most simple antenna array consisting of just two holes, spaced at *λ*. The small hole spacing of *λ* is chosen pragmatically, as this results in the lowest number of lobes in the radiation pattern, making it less likely to optimize to a local maximum in the optimization landscape. In this experiment every hole is illuminated with two beams at orthogonal polarization angle, for which both amplitude and phase are controlled independently. Therefore the optimization is done using not only phase but also amplitude and polarization state of each hole as optimization parameters. A covariance matrix algorithm (CMA) is used to obtain the illumination conditions that maximize the SPP intensity at the position of the near-field tip. The flexibility of our approach in obtaining directional SPPs is shown by positioning the near-field tip at 4 target location around the phased array. Each optimization was initiated using random starting conditions for the optimization parameters.

**Figure 4:**
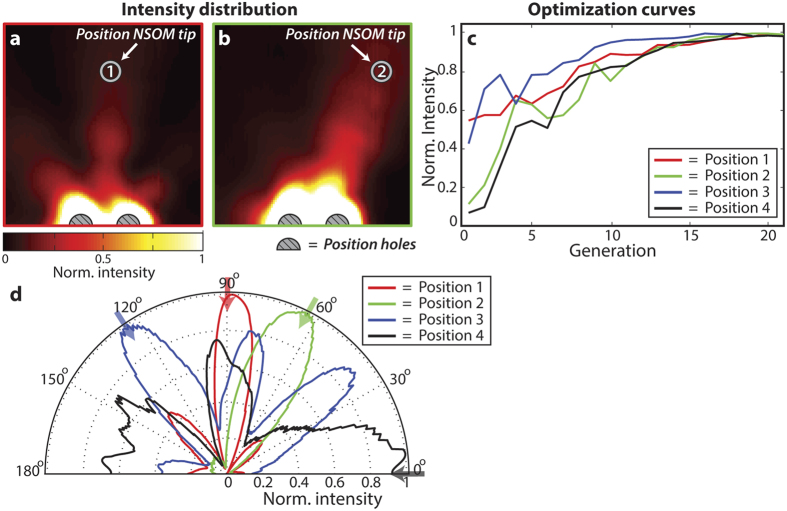
(**a**,**b**) The measured intensity distributions after optimization at position 1 and 2, indicated in the figure. (**c**) Evolution of the measured intensity at the optimization locations. (**d**) Polar plot of the angular intensity distribution after optimization. The arrows indicate the angle of the near-field tip with respect to the center of mass of the two holes.

Figure 4a,b show the SPP intensity distributions after an optimization has been performed at the near-field tip positions 1 and 2 (indicated with a circle). Both Fig. 4a,b show a directional beam of SPPs propagating towards the location of the near-field tip, leading to a strong local SPP field at the position of the near-field tip. Figure 4c shows the optimization of the intensity which is achieved at 4 near-field tip target locations. The optimization curves are normalized to their respective maximum value and generally approximate a maximum SPP intensity after 15 generations having 10 individuals. The optimized SPP intensity distributions at the 4 near-field tip positions are shown in the polar plots in Fig. 4. The arrows indicate the angle of the near-field tip with respect to the center of mass of the two holes. The angular distributions in Fig. 4d show that in all 4 cases the SPP intensity is successfully optimized at the location of the near-field tip, leading to angular distributions showing a strong peak in the direction of the near-field tip (indicated with the arrows).

## Discussion

Using compact phased antenna arrays consisting of just 2 or 5 elements, we have shown directional control of SPP radiation patterns. Directional control was achieved experimentally by controlling the illumination phase and polarization of each element of the phased antenna array. The control of the SPP radiation patterns was shown using two strategies. The first method, using rationally chosen illumination phase patterns, showed the relation between the illumination phase conditions of the hole arrays and the resulting SPP angular radiation patterns. The SPP radiation patterns can be well described using a simple dipole model, which is adopted from well known theory on phased antenna arrays. In the second method, the radiation patterns could be dynamically adjusted using a feedback-controlled optimization method using the signal detected by the near-field tip. This approach showcased the flexibility in obtaining directional control of the SPP radiation patterns.

Our results show the large potential of tailoring surface plasmons on the nanoscale by addressing individual holes. The obtained accurate control could be applied in tunable demultiplexing or “traffic control” in plasmonic circuits. Though the current implementation requires bulk optics for the phase controlled illumination of the holes, future approaches could use liquid crystals or phase change materials in or below the holes^34^. Additionally, by using a hole that has multiple transmission modes and out of plane polarization components, driving of the out-of-plane dipole moment of the hole becomes possible enabling directional emission of SPPs from single holes^35^,^36^.

## Additional Information

**How to cite this article**: Dikken, D. J. W. *et al.* A phased antenna array for surface plasmons. *Sci. Rep.* **6**, 25037; doi: 10.1038/srep25037 (2016).

## Supplementary Material

Supplementary Information
