# Supplementary material for: A phased antenna array for surface plasmons
Source: Sci Rep. 2016 Apr 28;6:25037. doi: 10.1038/srep25037 (PMC4848539; doi:10.1038/srep25037)
Supplement: Supplementary Information [file srep25037-s1.doc]

**­Supporting information with**

**“A phased antenna array for surface plasmons”**

Dirk Jan W. Dikken, Jeroen P. Korterik*,* Frans B. Segerink, Jennifer L. Herek and Jord C. Prangsma

**Fabrication:**

The hole structures where made using focused ion beam milling (FEI NOVA 600), which was done on a 250 nm thick gold film which was thermally evaporated on to a cover slip glass substrate. In order to improve the adhesion of the gold layer to the substrate a 0.5 nm thick chrome layer was used. Fabricated holes are designed having a diameter of 300 nm. The spacing of the holes presented in this article was designed to be 1 and 3 times the wavelength (0.95 µm and 2.85 µm)

**Illumination:**

The light source used is a Toptica DL pro tunable diode laser operating at 950 nm. The polarization in all experiments is with the illumination along the y-direction. The light is focused on the back side of the gold layer with a 0.9 NA objective. The illumination conditions (amplitude and phase) of the individual holes where controlled using a spatial light modulator (Pluto-NIR_015C) which is incorporated in a 4f design. Light impinging on every hole is associated to a specific area on the spatial light modulator, so that the illumination conditions for every hole can be set independently. The effective NA used for addressing each hole reduces with the number of holes. For addressing 5 holes an effective NA ≈ 0.4 per hole is estimated thus holes spaced by one wavelength are near the diffraction limit.

**Phase and polarization sensitive near-field scanning microscopy**:

An aperture based home built near-field optical microscope (NSOM) was used, which is capable of measuring the near-field amplitude, phase and polarization state [1,2,3] (see figure S1). The near-field phase is obtained by means of a heterodyne detection where the near-field microscope is incorporated in a Mach-Zehnder interferometer. The NSOM probe is coated with a 250 nm thick aluminum coating and has an aperture diameter of 225 nm [4].

A λ/2- and λ/4-plate in the signal arm are used to compensate the birefringence of the probe fiber. We exploit the fact that the free-space component of light which is transmitted through a sub wavelength hole maintains its polarization state to align the two λ-plates. For aligning the two λ-plates the near-field tip is positioned a couple of micrometer above a hole antenna which is illuminated with a well-defined linearly polarized beam of light. The λ-plates are positioned such that all the collected light is detected on one of the two detectors / lock-in amplifiers. This optimization procedure always leads to a SPP distribution which look similar to figure S2.

The measured signals at each position are L_x_ and L_y_, the complex output of two lock-in-amplifiers corresponding to the E_x_ and E_y_ polarized signal. The intensity (amplitude) of each hole is tuned by scanning over the holes and adapting the area used by the SLM to obtain equal amplitudes at each hole. We note here that it is currently accepted that an aperture NSOM detects both electric and magnetic field components. For the detection of surface plasmons on a gold film as we do here this is not of relevance as both E and H fields of a SPP are detected in the same polarization channel and are thus indistinguishable [5, 6].


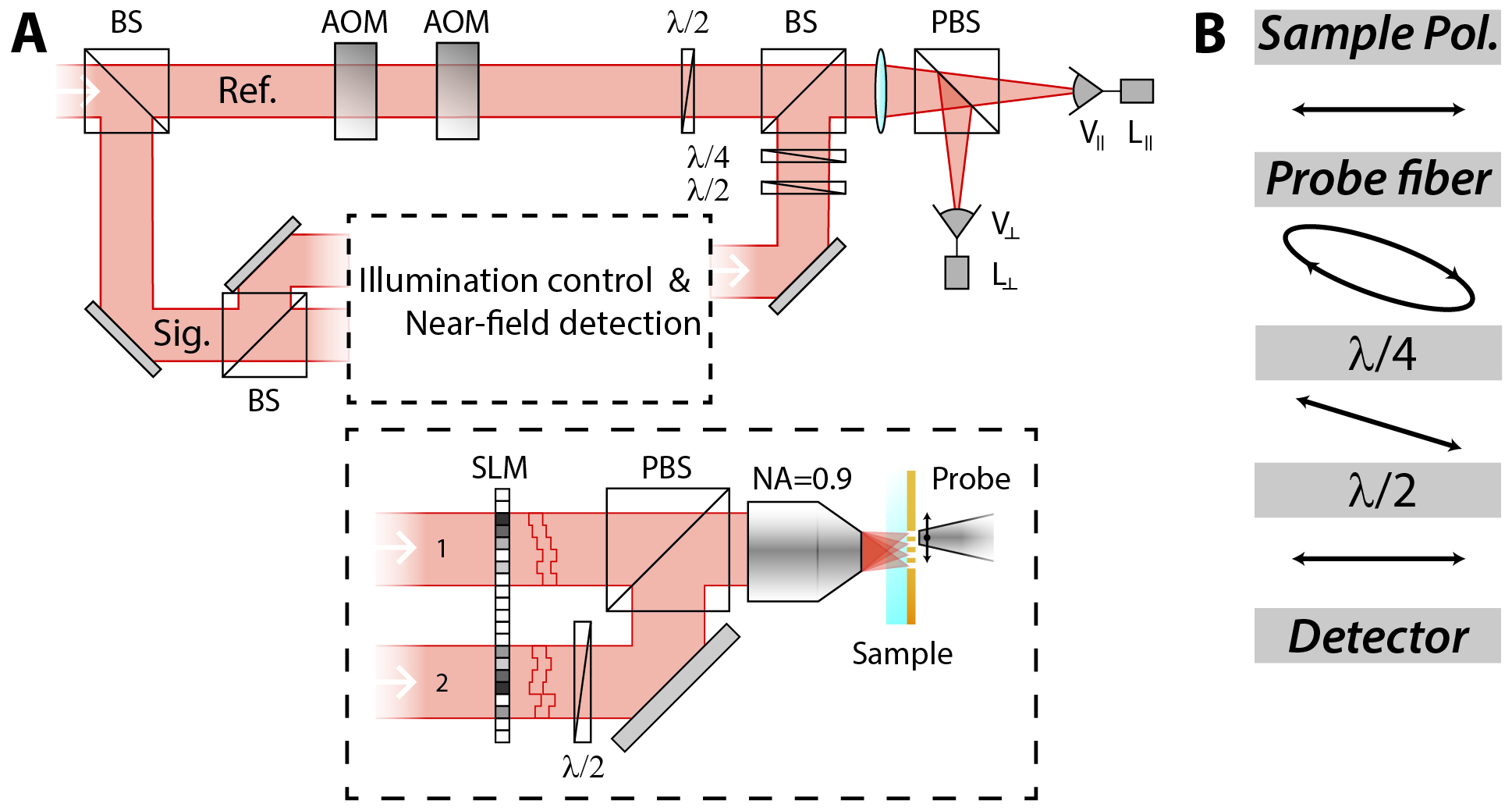


**Figure S1: Phase and polarization sensitive NSOM with spatial shaping.** (**A**) Light from a continuous wave laser (λ = 950 nm) is split into a signal and reference branch. The light in the reference branch is frequency shifted by 100 kHz using two AOMs. This is done in order to perform a heterodyne detection scheme using lock-in detection. A second Mach-Zehnder interferometer is created in the signal branch using a beam splitter cube. The phase front of the two resulting signal branches are shaped using a SLM, after which the second branch is passed through a half-wave plate (λ/2), which is set to rotate the polarization axis by 90^o^. The two beams are recombined using a polarizing beam splitter cube and directed into the objective. The sample is illuminated using a 0.90 NA air objective. The near-field distribution of the SPPs is collected using an aluminum coated aperture near-field probe, which is raster scanned over the sample surface. The light picked up by the near-field probe is coupled out to free-space, where after the signal is passed through a half- and a quarter-wave plate and recombined with the reference arm using a beam splitter. The combined signals from the signal and reference branch are impinging on the detectors (V) and are analyzed by a lock-in amplifier (L). A polarizing beam splitter cube enables the separate detection of the two orthogonal polarization components. In order to correctly map the orthogonal field components picked up by the near-field probe onto the two detectors (V_x_ and V_y_), the angles of the λ/4 and λ/2 in the signal branch are set to compensates for the birefringence of the fiber of the near-field probe (see **B**). The half-wave plate in the reference branch is used to distribute the reference light equally to both the detectors.

**Complex signals of a single hole.**

The near-field microscope setup, illustrated in figure S1, is used to obtain the field distribution of a single hole with a diameter of 300 nm. Both the parallel and orthogonal polarization components are detected simultaneously. The hole is illuminated with linearly polarized light, oriented in the vertical direction in figure S2 as well as all other figures in this paper. The λ/4 and λ/2 plates in the signal arm are set such that the experimental signals are linearly polarized and coincide with the vertical and horizontal polarization axis of the PBS before the detectors.


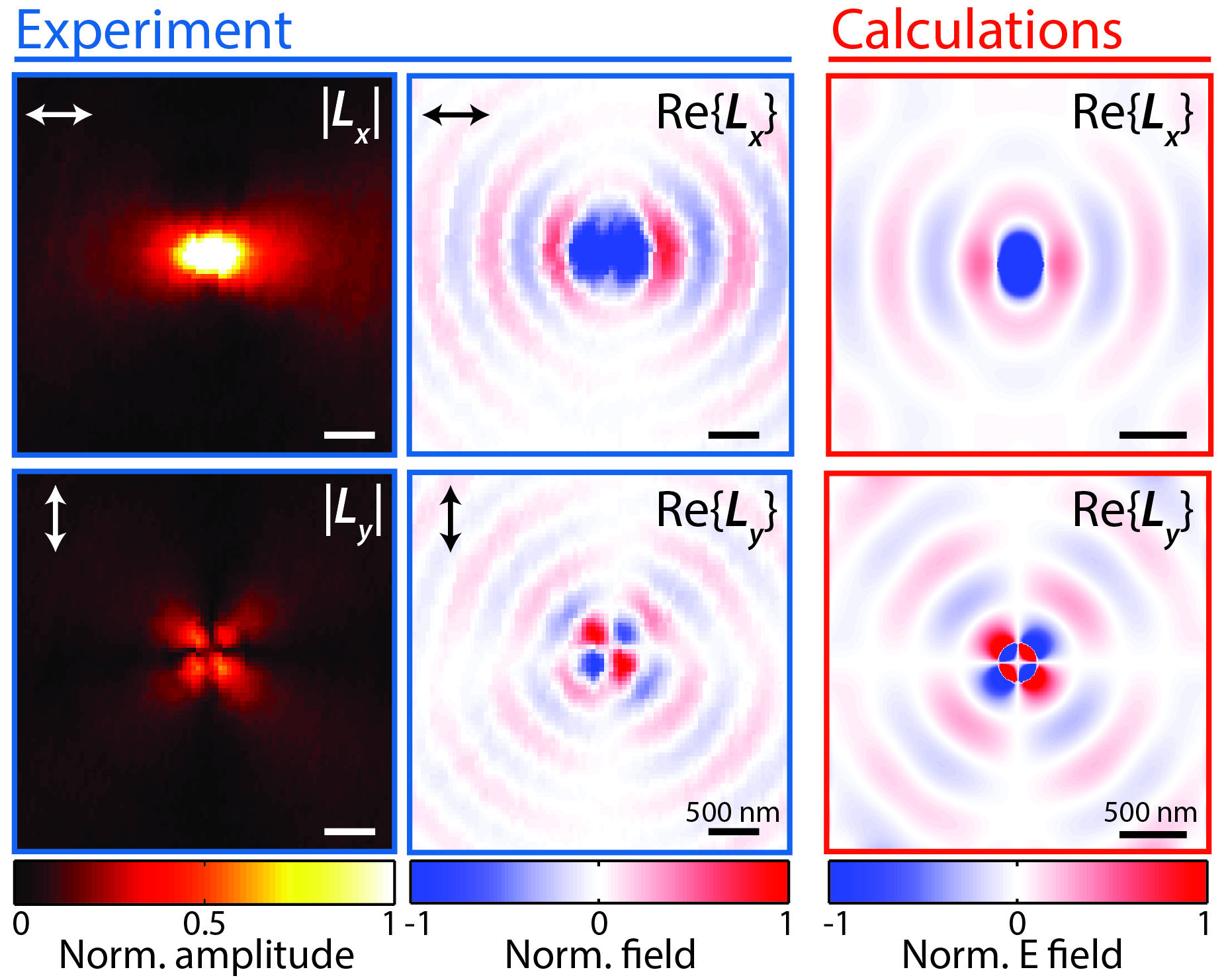


**Figure S2: Experimental and numerical field distributions of a single hole-antenna.** Parallel and orthogonal polarization components (indicated by the black and white arrows) of the field distribution of a single hole, with a diameter of 300 nm. The hole-antenna is illuminated with a linearly polarized beam which has a vertical polarization in this and all other images in this paper. Left and right show the absolute and real part, respectively, of the field distribution. The third row show real component of the complex fields, which are numerically calculated using a finite element method [7].

When the λ-plates are set correctly, the two obtained experimental signals closely resemble field distributions Re{E_x_} and Re{E_y_} that are calculated using a finite elements method [7]. The cross-polarized channel Lx, shows a twofold anti-symmetric four-lobed pattern. While the parallel polarized channel (Ly) shows a Gausian-like spot. It should be noted that the upward and downward propagating Ey (Ly) fields have the same phase, this is because we detect a longitudinal component of the SPP. If our near-field probe would be sensitive to the out-of-plane component (Ez) you would observe that upward and downward propagating SPPs are out-of-phase as would be expected from a dipole source.

**Dipole model of *N* coherent sources:**

The angle dependent emission of multiple coherently driven sources can be found in many standard text books on optics for instance [8]. Our only minor modification of this model is that, to incorporate the dipolar nature of the SPP emission, we add a cos^2^(θ) term to the equations. For completeness we included the derivation of this simple two dimensional model for the radiation pattern of N coherent sources. In the calculation of the angle dependent emission we assume the collection to be at a distant point *P* (in the far-field), so that the rays can be assumed parallel. The sum of the interfering dipolar sources at point P gives us an electric field which is given by the real part of:

$\tilde{E}=\cos(\theta)E_{0}(r)e^{-i\omega t}e^{ikr_{1}}\times\left[1+e^{i(k(r2-r1)+\alpha)}+e^{i(k(r3-r1)+\alpha)}+...+e^{i(k(rN-r1)+\alpha)}\right]$ (1)

The adjacent phase difference of the sources is defined as: , where *n* is the refractive index of the medium, *d* the spacing between sources and *α* is the hole to hole phase difference. Using this convention equation (1) can be rewritten to:


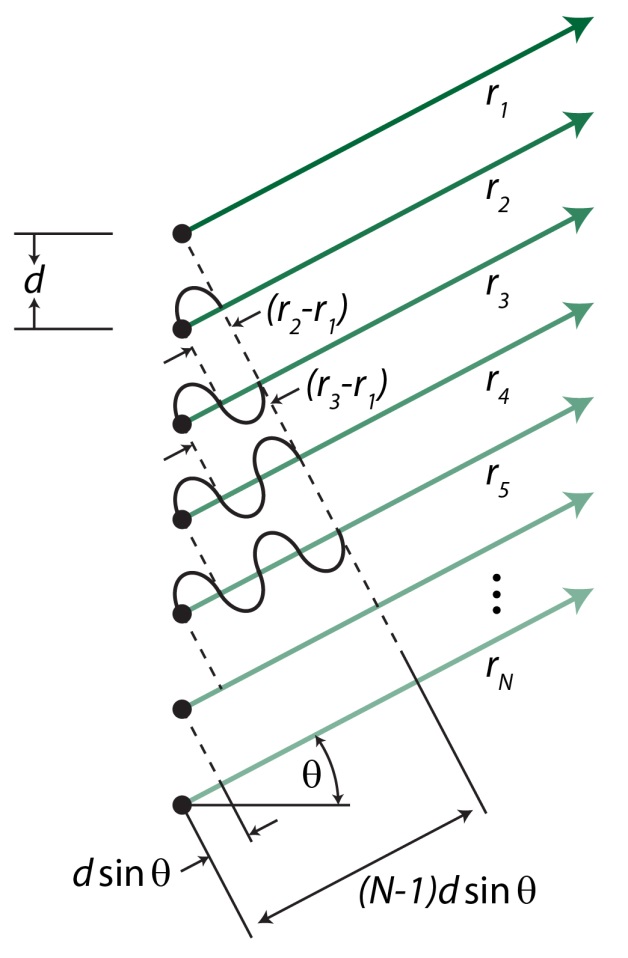
 (2)

where $\left[kr_{1}+(N-1)\delta/2\right]$ defines the distance from the center of the line of sources to point P.

The intensity diffraction pattern of the *N* sources can thus be written as :

$$I(\theta)=I0\cos^{2}(\theta)\frac{\sin^{2}(N\delta/2)}{\sin^{2}(\delta/2)}$$

**Figure S3: Array of coherent oscillators.** A linear array of coherent oscillators (*N*) which are spaced a distance *d* apart. This image was adapted from [8].

**Measurements with 2.85 µm spacing.**

The paper presents measurements in which the spacing is 950 nm. We also performed the same measurements on structures fabricated with 3 times larger spacing. Here we present these experimental results with the model results overlaid.


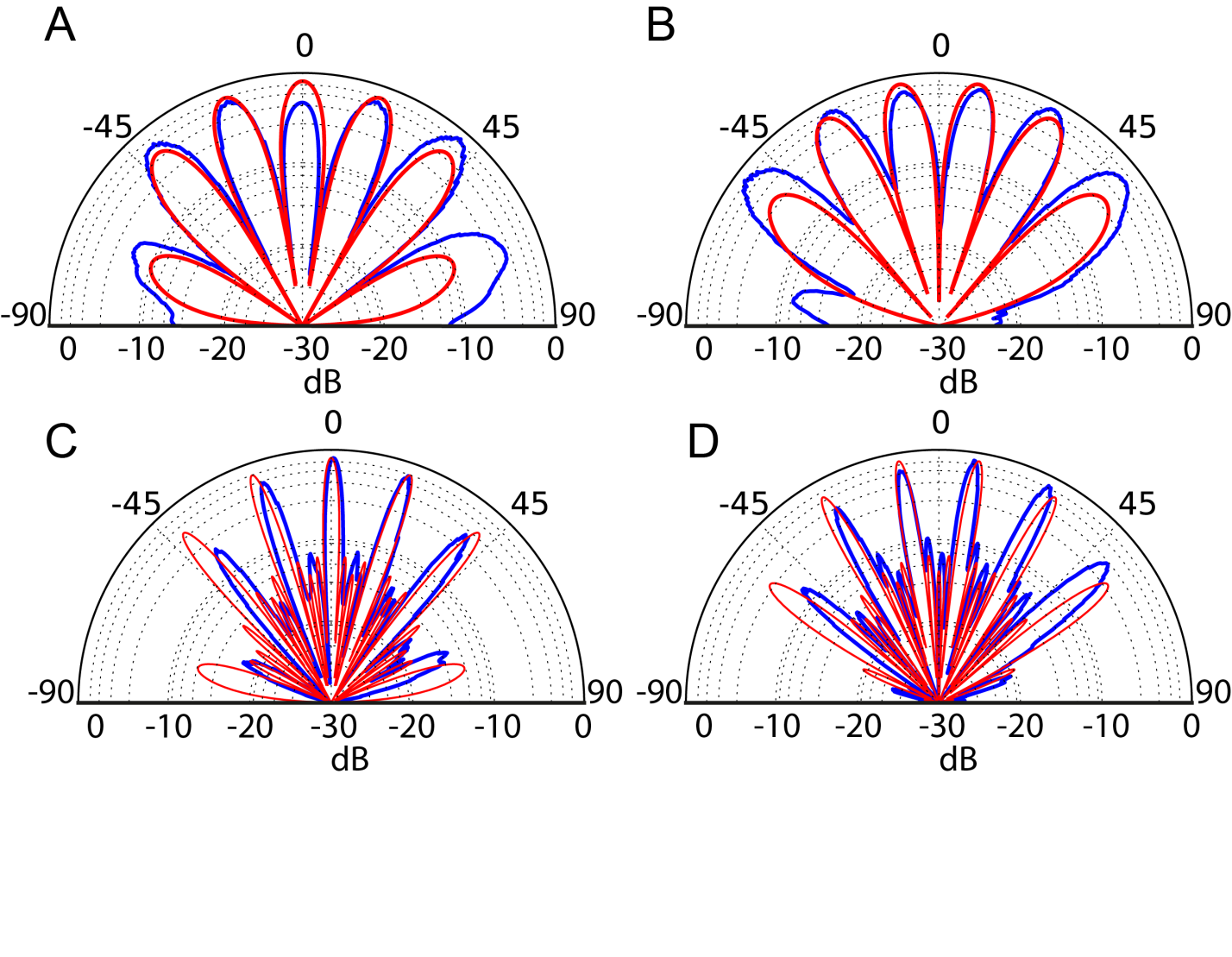


**Figure S4: Polar plots of angular radiation patterns of phased arrays consisting out of 2 and 5 elements.** The intensity radiation patterns measured (blue) and calculated (red) for a hole spacing of 2.85 µm. **A**, **B** and **C**, **D** are for phased arrays consisting of 2 and 5 hole-antennas, respectively. The holes in **A** and **C** are illuminated such that they are in-phase and **B** and **D** are illuminated such that the phase of sequential holes are half a cycle out-of-phase, The red lines are the modeled SPP radiation pattern using the simple model above.

**References:**

1. Balistreri, M. L. M.; Korterik, J. P.; Kuipers, L.; Van Hulst, N. F. *J. Light. Technol.* **2001**, *19*, 1169–1176.
2. Sandtke, M.; Engelen, R. J. P.; Schoenmaker, H.; Attema, I.; Dekker, H.; Cerjak, I.; Korterik, J. P.; Segerink, F. B.; Kuipers, L. *Rev. Sci. Instrum.* **2008**, *79*, 1–10.
3. Burresi, M.; Engelen, R.; Opheij, a.; van Oosten, D.; Mori, D.; Baba, T.; Kuipers, L. *Phys. Rev. Lett.* **2009**, *102*, 033902.
4. Veerman, J. A.; Otter, A. M.; Kuipers, L.; Hulst, N. F. Van. *Appl. Phys. Lett.* **1998**, *72*, 3115–3117.
5. Porto, J. A.; Carminati, R.; Greffet, J. J. *J. Appl. Phys.* **2000**, *88*, 4845–4850.
6. le Feber, B.; Rotenberg, N.; Beggs, D. M.; Kuipers, L. *Nat. Photonics* **2013**, *8*, 43–46.
7. CST microwave studio.
8. Hecht, E. *Optics*, 3^rd^ ed; Addison Wesley Longman, Inc, 1998
